# The lymphocyte immunophenotypical pattern in chronic lymphocytic leukemia associated with hepatitis viral infections

**Published:** 2011-08-25

**Authors:** H Bumbea, AM Vladareanu, A Vintilescu, S Radesi, C Ciufu, M Onisai, C Baluta, M Begu, C Dobrea, V Arama, A Streinu–Cercel, S Arama

**Affiliations:** * Hematology Department, Universitary Emergency Hospital, BucharestRomania; ** Victor Babes National Institute of Development and Research BucharestRomania; *** Matei Bals Institute of Infectious Diseases BucharestRomania; **** Carol Davila University of Medicine and Pharmacy , Pathophysiology Department, Bucharest Romania

## Abstract

**Background**: Chronic lymphoproliferative disorders (CLD) are frequently found in patients with hepatitis viral infections, which can lead to changes in pathogenesis. Hepatitis viruses are hepatotrope viruses, potentially lymphotrope and also potentially oncogenic (hepatocellular carcinoma) viruses. HBV and HCV are involved in autoimmune disorders and in the ethiopathogeny of chronic lymphoproliferative disorders.

**Aim**: Detection of immunophenotype changes of malignant lymphocytes in CLD – especially CLL – associated with hepatitis viral infections.

**Materials and methods**: Bone marrow aspirate, peripheral blood samples on EDTA were available for analysis from 58 patients from a follow–up schedule of the Department of Hematology SUUB from March 2008 until June 2009. The patients were diagnosed with chronic lymphoproliferative disorders associated with hepatitis virus B/C/D infections. A group of 28 consecutive unselected patients with CLL who met the diagnostic criteria of the National Cancer Institute–Working Group (NCI NCIWG), and associated hepatitis viral infection (v–CLL) were studied for the expression of several immunophenotypical markers, in comparison to CLL patients without viral infection (control group). Immunophenotyping analysis was performed on a FACS Calibur flowcytometer with a large panel according to EGIL/WHO recommendations. The diagnosis was completed after the histological and immunochemical analysis from tumoral lesions.

Results: demographics characteristics – male/female ratio 1/2, average age 64 years. Disease type: 90% B–CLD, 5% T–CLD, 5% Hodgkin's disease. The viral infections: 58,53% HCV, 34,41Z% HBV, 2,43% HBV+HDV, 2,43% HCV+HDV, 2,43% HBV+HCV+HDV. We found in CLL with viral coinfection (v–CLL) cases an elevated expression of B–cell markers – CD19 (Md95/92), CD20 (Md 90/39), CD79b (Md58/31), CD23 (Md67/37). Poor prognosis markers have a higher expression in v–CLL: CD38 (Md49/24), Bcl2 (Md 46/5), cyclin D19 (Md 11/0,5). No change in ZAP–70 expression was observed: Md 59,5/59,1.

**Discussions**: Hepatitis viruses could be involved in the pathogenesis of CLD, but as a trigger for a more aggressive outcome. Higher expression of B–cell markers CD19, CD20 in CLL with viral infection suggests a change to atypical CLL, sustained by elevated expression of known poor prognosis markers bcl–2, cyclin D1 and CD38. Lack of ZAP–70 expression could be explained by a strong correlation with a basic unmutated IgVH status, not related to the viral infection. We found a higher frequency of HCV infection in patients with CLD and especially in CLL patients, which were analyzed extensively for immunophenotypical changes. In the present study, we demonstrated that this CD5+ B cell population with clonal expansion, defining CLL patients, has a different immunophenotype, probably related to the hepatitis viral infection.

## Background 

### Hepatitic viruses

Hepatitis viruses are primary hepatotropic viruses and secondary lymphotropic. These viruses are potentially oncogenic, and they do have a role in development of hepatocellular hematoma [1,2,3]

Hepatitis B virus (HBV) is a DNA virus from Hepadnviridae family, which needs a reverstranscriptase to be able to replicate in infected cells genome. This mechanism is very similar to that of retroviruses. Primary tropism of these viruses is for hepatic tissue, but a tropism has also been demonstrated for other mononucleated cells (monocytes, B and T cells), and more uncommon, for neutrophils; these cells represent the extrahepatic reservoir for the virus.

Hepatitis C virus (HCV) is a RNA virus from Flaviviridae family, and has a mainly hepatic tropism. It has also the capacity to replicate in blood cells, especially in lymphocytes, and frequently produces neutropenia, thrombocytopenia, and mixed cryoglobulinemia. [4]

There is data which demonstrates that both HBV and HCV have a role in ethiopatogeny of autoimmune disorders and chronic lymphoproliferative disorders.[5,6] There has even been described a regional and demographic repartition [7], which sustains the association of viral hepatitis infections with occurrence of chronic lymphoproliferative disorders.

### Hepatitic viruses and chronic lymphoproliferative disorders

The arguments of this association between hepatitic viruses and chronic lymphoproliferative disorders are classified in  the following data categories:

- Epidemiologic and demographic
- Virusologic and molecular
- Pathogenic
- Targeted therapy impact

#### HBV infection and chronic lymphoproliferation syndromes

HBV can replicate in extrahepatic tissues–bone marrow, lymph nodes, and may be involved in extrahepatic pathology. The presence of ‘aberrant’ non–replicative HBV–DNA or viral antigens in mononucleated blood cells (monocytes, B cells, CD4 and CD8 T cells) suggests an aberrant replication and transcription and sustains the idea of an extrahepatic reservoir with chronic stimulation of lymphocytes and clonal transformation in further period.

It has been suggested that HBV reactivation may appear even in patients in whom a viral clearance  was obtained after an acute infection with HBV, thus the usage of more potent cytostatics induce a higher risk to viral reactivation.[8,9]

#### The role of HBV in lymphomagenesis

Many articles showed that the risk of occurrence of non–Hodgkin lymphoma (NHL) is 2–3 times higher in patients with HBs antigen portage, than those with negative serum HBs antigen – Table 1. Thus, Liang [10] reported a prevalence of 22% of HBV infection in 484 patients with NHL in Hong Kong. Concerning the age of patients, a higher number of NHL was noticed in young people with HBV. Kim JH [11] reported in 2002 a high risk of B–cell NHL occurrence in HBs antigen portage individuals. 

**Table 1:** Increased incidence on NHL in patients with HBV

Country	Number of patients with NHL	HBV Seroprevalence	Author, year
Korea	222	12,6%	Kim, 2002
Japan	348	6,9%	Kuniyoshi, 2001
Hong–Kong	484	22%	Liang, 1990

The hypotheses of association of HBV infection with NHL are the following:

- The risk of infection or viral reactivation is increased because of direct immunosuppressive effect of lymphoma
- HBV is directly involved in lymphomagenesis through chronic antigenic stimulation
- Implication of an unknown virus which has a similar transmission path as HBV

#### HVC infection and chronic lymphoproliferative disorders

HCV prevalence in patients with NHL with B cell or other chronic lymphoproliferative disorders is between 8 and 32%, depending on the author.[12]

HCV is a hepatotrope virus, but it may infect and replicate in hematopoietic cells, too [13,14]. The presence of HCV RNA was also demonstrated in B, T cells and monocytes of patients with HCV infection. 

Proliferation of B cell clone occurs mainly in bone marrow and liver, as a response to the chronic stimulation of HCV virus antigens, E2 protein, respectively, and this clone has a risk for malignant transformation [15]

Protein E2 contains the link site with a membrane glycoprotein on the surface of hepatocyte and lymphocytes, named **CD81**= specific receptor for HCV of hepatocytes and B cell.

A high risk for developing NHL is represented by the presence in peripheral blood B cells of a translocation which involves the antiapoptotic gene bcl–2, t(14;18), respectively.

The arguments are especially sero–epidemiological, represented by the presence of antibodies against HCV in serum and of HCV RNA in tumoral lymphomatous cells.

At the same time, we may regard HCV as one of the possible etiologic agents of lymphomas, considering the association between HCV infection and essential mixed cryoglobulinemia (EMC), in relationship with the hypothesis that type II EMC could represent a low grade malignant lymphoma.

Involvement of HCV in lymphoproliferative disorders is a problem of current interest, but still controversed. Considering the HCV lymphotropism and the hypothesis that infection of B cells with HCV produces a monoclonal proliferation, the theory of HCV involving in B cell NHL pathogenesis has been produced.

The epidemiologic association of NHL and chronic infection with HCV (Table 2) The association between HCV infection and B cell NHL was first described in 1994 by Ferri C [16]:

- 32% of B cell NHL patients have serum and virusological markers of HCV infection; HCV RNA have been identified even in serum and peripheral lymphocytes.
- Frequently, patients with B cell NHL have also EMC

Association of HCV infection with T cell NHL and Hodgkin disease does not seem evident, since in Hodgkin Lymphoma, the infection with HCV was reported only in 5% of patients. 

Successive studies done by Zignego et al in 1995 [17], 1996 [18] and 1997 detect HCV viremia in 35% of patients with B cell NHL, the most frequent genotypes 1b (61% of cases) and 2a (14% of cases). HCV was found more frequently in intermediary aggressive malignant lymphomas. 

Other studies [19,20,21] do not detect the presence of a higher prevalence of HCV infection in NHL patients. These authors suggest that the association of NHL with HCV infection is strictly related to the geographic distribution of HCV infection prevalence, and to different viral types and subtypes (Table 2).

**Table 2:** Prevalence of HCV infection in patients with NHL – adapted

Country	Patients with NHL (no)	Prevalence of HCV infection in NHL patients (%)	Controls (no of patients)	Prevalence of HCV infection in controls	Author, year
USA	120	22	114	5	Zukermann,1997
USA	312	11,5	ND	ND	Kashyap,1998
Italy	199	28,6	6,917	2,87	Mazzaro,1996
Italy	115	32	70	0	Zugnega,1995
Italy	175	37	175	10	Vallisa,1999
Italy	91	23	1568	1,9	De Rosa,1997
Japan	54	22,2	ND	ND	Izumi,1996
Brasil	109	9	98	2	Chindamo,2001
Egypt	227	42	227	ND	Cowgill, 2004
Italy	400	17,5	ND	ND	Mele,2003
Japan	348	8,1	ND	ND	Kuniyoshi,2001

Dal Maso [23] shows in 2006 a meta–analysis in which he evaluates the intensity of association between HCV infection and non–Hodgkin lymphomas (NHL); he identified a significant relative high risk for all major subtypes of B cell and T cell NHL.

## Materials and methods

- Correlation of diagnosis methods of evaluation of hepatitic viruses involvement in chronic lymphoproliferative disorders – infection prevalence and identification of some molecular mechanisms involved in oncogenesis
- Prospective analytical observational study – part of LIMFOVIR project –multidisciplinary research of molecular mechanisms involved in chronic lymphoproliferative disorders genesis

### General Objectives

1. Positive diagnosis of chronic lymphoproliferative disorders
2. Immunophenotypic and histopathologycal differential diagnosis based on WHO classification
3. Statistic analysis of clinical and paraclinical changes
4. Identifying of correlations between these parameters and prognosis subsets

### Special Objectives 

#### Initial steps

1. Patient population: There were 58 patients enrolled from follow–up schedule of Department of Hematology Emergency University Hospital Bucharest, from March 2008 until June 2009, diagnosed with chronic lymphoproliferative disorders associated with hepatitis virus B/C/D. A group of 28 consecutive, unselected patients with CLL who met the diagnostic criteria of the National Cancer Institute–Working Group (NCI NCIWG), and viral coinfection were studied for the expression of several immunophenotypical markers. These patients were studied in detail by analyzing the following parameters measured when putting the diagnosis or during the follow–up: lymphocyte and platelet counts; lymphocyte doubling time (LDT); hemoglobin (Hb), immunoglobulin and β–2–microglobulin serum levels; liver and spleen enlargement; size of lymph nodes involved; extranodal localizations; autoimmune manifestations; disease stage according to Rai modified criteria [24]; history of treatment; and progressive or stable disease as defined by the NCI–WG.
2. Filling in patients monitoring files (CRF), including clinical, biological and hematological data: general epidemiological data: sex, age, region; epidemiological viral infection data; lymphoproliferation type  and viral infection type; treatment: chemotherapy, antiviral drugs; clinical parameters; biological parameters; immunophenotypic parameters.
3. Correlation of information from monitoring files (CRF) with database proceedings associated to the entire project.

#### Immunophenotyping technics

Immunophenotypic analysis was performed on fresh blood samples (PB) and bone marrow aspirate (BM). In individual cases the analysis was concomitantly performed on both PB and BM. 

We used a four colors flowcytometer BD FACS Calibur, with two lasers, main argon laser with emission in 488 nm, and the secondary red emission in 613 nm. The following fluorochromes were used: FITC –FL1, PE, RD1 – FL2, PC5, PerCP – FL3, APC – FL4; the monoclonals were standard conjugated by the producer: Coulter, Immunotech, Becton–Dickinson, BD Pharmingen, Dako.

### Surface Staining

1. the sample was collected as peripheral blood by endovenous punction, or bone marrow aspirate; the blood was introduced in standard vacuteiner with anticoagulant (EDTA); the samples were stored at room temperature
2. the sample was adjusted at 100000 cells on μl, and 100 μl of sample was introduced in each Falcon tube used for acquisition
3. the staining was done with 5–20 μl of monoclonals per tube
4. the stained tube was incubated 15 minutes in dark, at room temperature
5. erythrocyte lysis and cellular membrane fixation with FACS Lyse Solution (Becton–Dickinson) 500 μl
6. analysis in flowcytometer after 10–15 minutes
7. the analysis of sample was done with isotypic control for each fluorescence in order to have in the double negative quadrant at least of 98% of events
8. analysis of samples

### Intracellular staining

For permeation and study of intracellular markers, we used the standard protocol for IntraPrep, recommended by the producer and presented below:

- Staining for surface antigens
- Fixation of cellular membrane with Reagent 1, 100 μl/tube, incubation (dark, room temperature, 5 minutes)
- Washing with PBS (Phosphate Buffer Solution), centrifugation at 2000 rotations/minute, release supernatant
- Permeation with Reagent 2, 100 μl/tube, incubation (dark, room temperature, 5 minutes)
- Washing with PBS, centrifugation at 2000 rotations/minute, release supernatant
- Staining with monoclonal antibody for intracellular antigen
- Incubation (dark, room temperature, 15 minutes)
- Washing with PBS and centrifugation at 2000 rotations/minute, release supernatant
- Adding PBS 500 μl / tube
- Acquisition in the flowcytometer.

Panel of monoclonal antibodies: We used for immunophenotyping analysis the following monoclonals for surface staining: CD19, CD20, CD45, CD43, CD79b, CD5, CD23, CD24, CD38, CD11a, CD10, IgM, IgG, IgD, CD81, CD103, FMC7. The intracellular staining was done for following markers: cyclin D1, bcl–2, ZAP–70.

## Results

### General epidemiological data

Our patient group comprised 58 patients diagnosed with chronic lymphoproliferative disorders (CLD), with a male/female ratio of 1/2.

**Figure 1:**
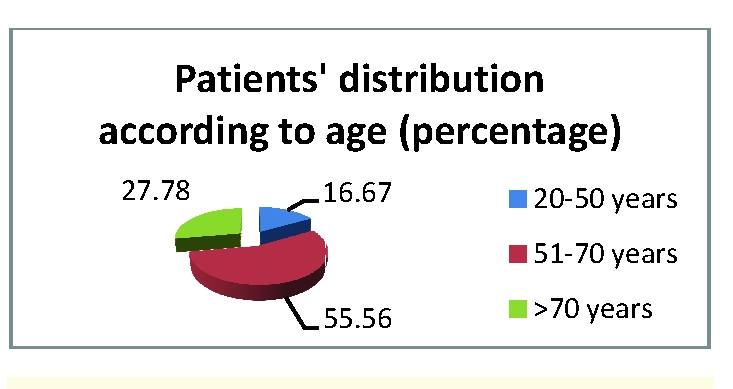
Distribution of CLD patients according the age–patients group

We stratified the group on age criterion into three groups, and we found the medium age of 64 years, with most of the patients between 51 and 70 years old –more than 55% of cases – Figure 1

### Epidemiological data concerning the viral infection and the CLD type

**Figure 2:**
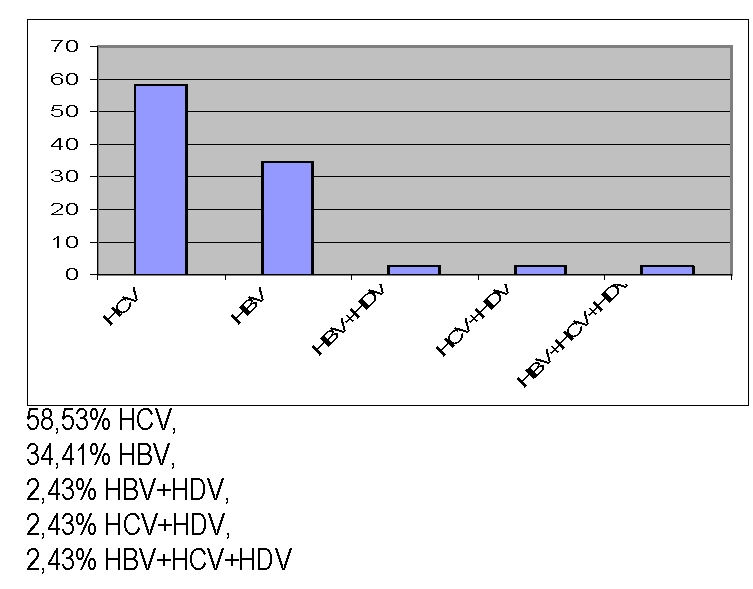
Prevalence of hepatitis viruses' infection – patients group; 58,53% HCV, 34,41% HBV, 2,43% HBV+HDV, 2,43% HCV+HDV, 2,43% HBV+HCV+HDV

The majority of the patients presented HCV infection; we also noticed that coinfections were also present – even with 3 viruses at the same time (2.43%)–Figure 2

**Figure 3:**
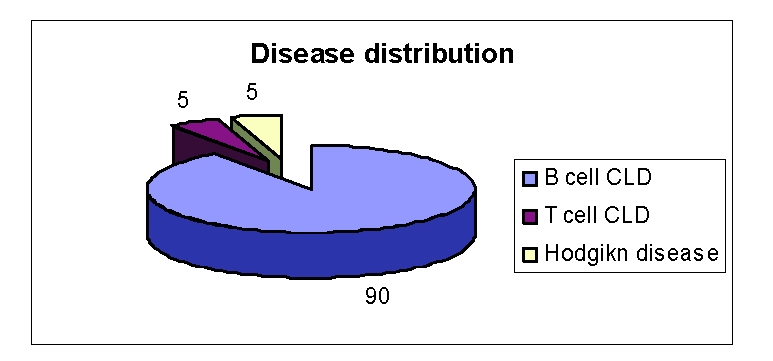
Type of lymphoproliferative disorder – patients group; 90% B–CLD; 5% T–CLD; 5% Hodgkin disease

Most of the patients presented B–cell chronic lymphoproliferative disorders (over 90%)– Figure 3

Regarding the clinical parameters, we assessed the presence of B signs (55,5% of patients), lymphadenopathies (61% of patients), hepatosplenomegaly (55.5% of patients), signs of liver failure (such as: presence of jaundice, ascitis, portal hypertension, hemorrhagic complications– total 11% of patients)

Hematological parameters are presented in Table 3.

**Table 3:** Hematological parameters of patients with CLD and hepatitis viral infection

	Hematological evaluation
Hb (results are presented as: median (minimum value, maximum value) (g/dl)	12.1 (8.5–14.7)
Platelet count(/mm)	180000 (27000–571000)
Lymphocytosis	39%
Medullar involvment	50%

Cryoglobulinemia was found in 5 patients, all of them with HCV coinfection, and in 1 patient was found autoimmune hemolytic anemia. 

Liver damage was assessed by the following parameters: elevated levels for serum AST, ALT (10 patients); liver cholestasis (3 patients), and presence of coagulopathy – prolonged coagulation times (4 patients).

#### Immunophenotyping

We established two main groups of patients analyzed: CLD without viral infections and v–CLD with one or more viral infections. We performed a more detailed analyze of a subset of CLD, the patients with chronic lymphocytic leukemia (CLL), respectively, because of the significant percentage those patients represented from all CLD group–Table 4

**Figure 4:**
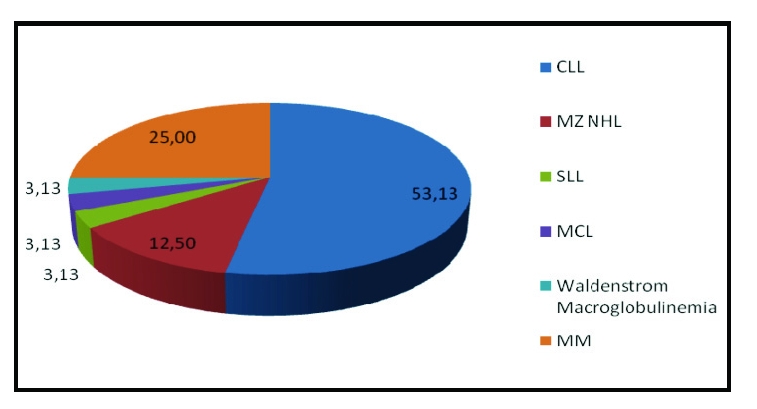
Type of B–cell lymphoproliferative disorders; Legend: CLL –chronic lymphocytic leukemia, SLL – small–cell lymphocytic lymphoma, MZ–NHL – marginal zone non–Hodgkin lymphoma, WM – Waldenstrom's macroglobulinemia, MCL – mantle cell lymphoma, MM – multiple myeloma

Out of 58 patients with v–CLD, we selected 28 patients with CLL and viral infection with at least one hepatitis virus (group vZ–CLL). This group of patients was analyzed in comparison to the similar patients diagnosed with CLL, but without any documented viral infection (group CLL).

#### Statistical analysis

We used SPSS 16.0, Windows software for statistical analysis.

The expression of specific markers was analyzed as mean fluorescence intensity (MFI).The analysis was performed on all markers used to identify lineage markers for B cells and prognosis or activation markers. We compared the median of values found (Md). The results are shown in the Table 4.

#### CD19 and CD20

**Figure 5:**
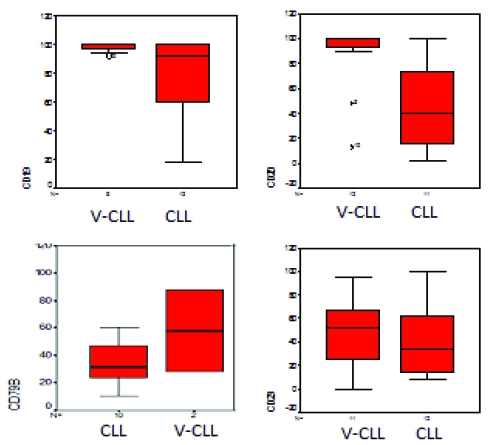
Statistical representation (box–plot) of expression of CD19, CD20, CD79b and CD23 in CLL and V–CLL groups of patients. Analysis performed with SPSS software.

The expression of lineage markers for CD19 was found at a Md value of 92 in CLL group and 95 in v–CLL group – Figure 5)(p>0.05). For CD20, Md was 39 in CLL group and 90 in v–CLL group – Figure 5)(p=0.004). It is known that usually malignant B–CLL cells express a low intensity of CD20, therefore the increasing value obtained in v–CLL is very significant. Because the level of CD19 expression is not so diminished as CD20, the increase is lower for CD19 than CD20 in v–CLL group.

**Table 4:** immunophenotypical markers in patients with CLL, with or without associated viral infection

CLL patients	CD19 (Md)	CD20 (Md)	CD23 (Md)	CD79b (Md)	CD38 (Md)	ZAP–70 (Md)	Cyclin D1 (Md)	Bcl–2 (Md)

#### CD23

Expression of CD23 was found at a Md value of 37 in MFI in CLL group without viral infection – Figure 5 (p>0.05). This marker could be characterized as an activation marker in normal B cells. In our v–CLL group of patients, the values were at a Md of 67 in MFI, significantly increased compared to the patients without viral infection.

#### CD79b

CD79b is usually low or negative in CLL patients, and it is an important marker used in positive diagnosis of CLL. We found also an increase of this marker in CLL patients with viral infections (v–CLL) – Md value of MFI of 58 versus 31 in non–infected CLL patients – Figure 5 (p=0.04).

#### CD38, ZAP–70, bcl–2 and cyclin D1

**Figure 6:**
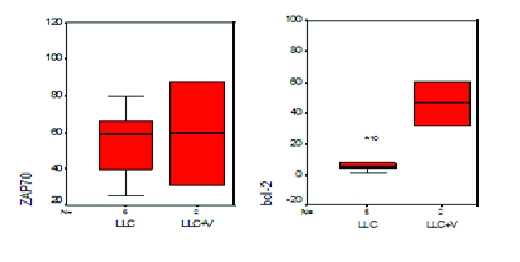
Statistical representation (box–plot) of expression of ZAP–70 and bcl–2 in patients with CLL and V–CLL. Analysis performed with SPSS software

Prognosis markers for CLL were also analyzed in our patient groups. We found an increased value in CD38, bcl–2 and cyclin D1 markers in patients with viral infection, but unfortunately without statistical significance (p>0.05). On the other hand, ZAP–70 expression was not different between the two groups – Figure 6 .

Surrogate markers for progression of disease were not found different between the two compared groups, which suggests that the viral coinfection does not interfere with the somatic hypermutation of heavy chains of immunoglobulin.

**Table 5:** immunophenotypical markers in patients with CLL, with or without associated viral infection

CLL patients	CD19 (Md)	CD20 (Md)	CD23 (Md)	CD79b (Md)	CD38 (Md)	ZAP–70 (Md)	Cyclin D1 (Md)	Bcl–2 (Md)
Without viral infection	92	39	37	31	24	59.1	0.5	5
With viral infection	95	90	67	58	49	59.5	11	46

## Discussion

Chronic hepatitis viral infection is associated with a multitude of extrahepatic manifestations, including autoimmune phenomena, benign clonal expansion of B cells, and B–cell non–Hodgkin lymphoma, which suggest B–cell activation and proliferation. However, the exact mechanism linking HBV or HCV infection with autoimmunity and lymphoproliferation is unknown. For HCV, the interaction between the HCV envelope 2 glycoprotein of HCV and the CD81–containing complex on B cells may provide one of these missing links. Of particular interest are the activation and proliferation of a specific B–cell subpopulation, namely, the CD5+ cells [25]. These cells proliferate in patients with essential mixed cryoglobulinemia and various other autoimmune disorders [25,23,27,28,29]

We noticed a lower average age in CLL patients with hepatitis viral coinfection than the average age for CLL in literature. We found out from our study a higher frequency of HCV infection in patients with CLD, and especially in CLL patients, which were analyzed extensively for immunophenotypical changes. In the present study, we demonstrated that this CD5+ B cell population with clonal expansion, defining CLL patients, has a different immunophenotype related to the hepatitis viral coinfection. Some studies showed that B-cell proliferation in HCV–infected patients is probably enhanced by HCV–specific properties, including the ability of HCV proteins to bind to CD81 on the B–cell surface and to influence intracellular regulatory functions following viral entry into B cells. Additionally, there is recent data indicating that HCV infection and clonal expansion of B cells within the liver preferentially involve RF–producing cells [30] and that in selected cases of patients with type II mixed cryoglobulinemia, B–cell subsets expressing IgM RF are the prevalent cell type targeted by HCV [31]. Based on these data, it is reasonable to speculate that CD5+ B–cell activation and proliferation are facilitated by HCV binding to the cell surface–associated CD81–containing complex. In support of this hypothesis is the recent evidence showing that CD81 on B cells plays an important role in generating antibody responses to T–cell–dependent Th2 antigens [32]. 

Our study demonstrates that hepatitis viruses are not only involved in clonal expansion and genesis of lymphoproliferative disoders, but there are important changes in the usual immunophenotype of clonal CD5 cells in CLL patients. High expression of CD19, CD20 and CD79b in patients with viral coinfection suggests an atypical immunophenotype. Furthermore, expression of bcl–2 and cyclin D1 is overexpressed in patients with an atypical CLL [33,34] and it was found also in our patients with viral coinfection. These proteins are involved in apoptosis inhibition and could be related with the resistance to treatment in these patients.

Increased expression of CD38 and CD23 found in CLL patients with viral coinfection could be correlated with activation of B cell, possibly because of antigenic viral stimulation.

Surrogate markers for progression of disease as ZAP–70, were not found changed, which suggests that the viral coinfection does not influence the somatic hypermutation of heavy chains of immunoglobulin. It is known that these markers are related more to the risk for progression in CLL patients [35]. These findings [36] suggest that viral coinfection does not induce a risk for progression in CLL patients, but a higher risk of resistance to treatment.

## Conclusions

The involvement of hepatitis viruses in lymphomagenesis is a very exciting field and it is still in debate. The analysis of our patients in this first part of the study in project LIMFO–VIR was done in patients with chronic lymphoproliferative disorders associated coinfection with B, C, D hepatitis viruses. Our results suggest that the coinfection appears more frequent in patients older than 50 years, with higher frequency in women.

The most frequent virus was HCV, associated mostly with indolent type CLDs, with extranodal disease, splenomegaly, lymphocytosis, cryoglobulinemia and liver damage.

Preliminary data shows a significant change in CLL immunophenotype in patients with viral coinfection, which suggests transformation to a more aggressive disease, based on the expression of a lymphoma–like immunophenotype. Because of overexpression of antiapoptotic proteins and no change of surrogate markers for IgVH somatic hypermutation status, we speculate that viral infection could induce a more resistant malignant clone, but not a higher risk for progression in patients with viral coinfection and CLL.

Our results could be important in assesement and strategy of therapy in patients with viral infectious and lymphoproliferative disorders.
